# Optimal combination of heart and lung dose parameters in radiotherapy for locally advanced non-small cell lung carcinoma: a multicenter retrospective study

**DOI:** 10.1093/jrr/rrag034

**Published:** 2026-05-14

**Authors:** Tomohiko Miyazaki, Koichi Yasuda, Hiroshi Taguchi, Hideki Minatogawa, Tetsuya Inoue, Rikiya Takashina, Manami Otsuka, Keiji Nakazato, Noriaki Nishiyama, Keiji Kobashi, Izuru Otake, Jun Sakakibara-Konishi, Satoshi Oizumi, Hajime Kikuchi, Takayuki Hashimoto, Hidefumi Aoyama

**Affiliations:** Department of Radiation Oncology, Graduate School of Medicine and Faculty of Medicine Hokkaido University, North 15 West 7, Kita-ku, Sapporo, Hokkaido 060-8638, Japan; Department of Radiation Oncology, Graduate School of Medicine and Faculty of Medicine Hokkaido University, North 15 West 7, Kita-ku, Sapporo, Hokkaido 060-8638, Japan; Department of Radiation Oncology, Hokkaido University Hospital, NHO Hokkaido Cancer Center and Obihiro Kosei Hospital, 2-3-54, Kikusui-4, Shiroishi-ku, Sapporo, Hokkaido 003-0804, Japan; Department of Radiation Oncology, Hokkaido University Hospital, NHO Hokkaido Cancer Center and Obihiro Kosei Hospital, 2-3-54, Kikusui-4, Shiroishi-ku, Sapporo, Hokkaido 003-0804, Japan; Department of Radiology, Obihiro Kosei Hospital, West 15, South 10, Obihiro, Hokkaido 080-0024, Japan; Department of Radiation Oncology, Graduate School of Medicine and Faculty of Medicine Hokkaido University, North 15 West 7, Kita-ku, Sapporo, Hokkaido 060-8638, Japan; Department of Radiation Oncology, Hokkaido University Hospital, NHO Hokkaido Cancer Center and Obihiro Kosei Hospital, 2-3-54, Kikusui-4, Shiroishi-ku, Sapporo, Hokkaido 003-0804, Japan; Department of Medical Physics, Hokkaido University Hospital, North 14 West 5, Kita-ku, Sapporo, Hokkaido 060-8648, Japan; Department of Radiation Oncology, Hokkaido University Hospital, NHO Hokkaido Cancer Center and Obihiro Kosei Hospital, 2-3-54, Kikusui-4, Shiroishi-ku, Sapporo, Hokkaido 003-0804, Japan; Global Center for Biomedical Science and Engineering, Faculty of Medicine, Hokkaido University, North 15 West 7, Kita-ku, Sapporo, Hokkaido 060-8638, Japan; Department of Radiation Oncology, Graduate School of Medicine and Faculty of Medicine Hokkaido University, North 15 West 7, Kita-ku, Sapporo, Hokkaido 060-8638, Japan; Department of Respiratory Medicine, Hokkaido University Hospital, North 14 West 5, Kita-ku, Sapporo, Hokkaido 060-8648, Japan; Department of Respiratory Medicine, Hokkaido University Hospital, NHO Hokkaido Cancer Center and Obihiro Kosei Hospital, 2-3-54, Kikusui-4, Shiroishi-ku, Sapporo, Hokkaido 003-0804, Japan; Department of Respiratory Medicine, Obihiro Kosei Hospital, West 15, South 10, Obihiro, Hokkaido 080-0024, Japan; Global Center for Biomedical Science and Engineering, Faculty of Medicine, Hokkaido University, North 15 West 7, Kita-ku, Sapporo, Hokkaido 060-8638, Japan; Department of Radiation Oncology, Graduate School of Medicine and Faculty of Medicine Hokkaido University, North 15 West 7, Kita-ku, Sapporo, Hokkaido 060-8638, Japan

**Keywords:** radiation oncology, locally advanced non-small cell lung carcinoma, multicenter retrospective study, heart dose, cardiac toxicity, overall survival

## Abstract

This study aimed to identify the optimal combination of heart and lung dose parameters associated with overall survival (OS) in patients with locally advanced non-small cell lung carcinoma (LA-NSCLC) receiving radiotherapy. Data from 278 patients treated definitively for LA-NSCLC at three institutions between 1 April 2013 and 31 October 2021 were retrospectively analyzed. Lung and heart dose parameters were categorized into high- or low-dose groups based on predefined thresholds for subsequent analysis. Univariable analysis of OS was conducted for all dose group combinations. The combination with the lowest *P*-value was identified as the most promising and included in the multivariable analysis along with other clinical factors. The combinations of mean heart dose (MHD)/lung V40 (LV40) (‘Vxx’ denotes the percentage of organ volume receiving ≥xx Gy) and heart V5 (HV5)/LV40 yielded the lowest *P*-values (*P* = 0.021). The thresholds for these dose parameters were MHD ≤24.4 Gy, HV5 ≤63% and LV40 ≤7.7%, respectively. Multivariable analysis incorporating clinical factors identified age ≥65 years (HR: 2.06, *P* = 0.002), performance status ≥1 (Hazard ratio [HR]: 1.68, *P* = 0.024), chemoradiotherapy (HR: 0.28, *P* < 0.001), current smoking history (HR: 2.21, *P* < 0.001), gross tumor volume (HR: 1.43, *P* = 0.007) and the MHD/LV40 combination (HR: 2.35, *P* = 0.003) as independent prognostic factors. While exploratory, this study highlights the clinical significance of the MHD/LV40 combination as a prognostic factor; integrating these parameters radiotherapy for LA-NSCLC may improve patient outcomes.

## INTRODUCTION

Although concurrent chemoradiotherapy (CRT) remains the standard definitive treatment for locally advanced non-small cell lung carcinoma (LA-NSCLC), treatment outcomes continue to be unsatisfactory [1]. The addition of durvalumab, an immune checkpoint inhibitor, as maintenance therapy has significantly improved overall survival (OS) and progression-free survival (PFS), representing a major advancement in the prognosis of LA-NSCLC. This approach is now widely accepted as the standard of care [2, 3]. Regarding radiotherapy (RT) optimization, the Radiation Therapy Oncology Group (RTOG) 0617 trial—a prospective phase III study examining the efficacy of dose escalation—demonstrated no prognostic benefit in the high-dose group (74 Gy). As a result, 60 Gy has remained the standard radiation dose for CRT in LA-NSCLC [4]. A secondary analysis of RTOG 0617 suggested that several heart dose parameters are independent prognostic factors for OS [4], drawing increasing attention to cardiac toxicity in recent years [5–8]. Extensive knowledge exists regarding the pulmonary toxicity of RT for lung cancer, particularly radiation pneumonitis (RP). Dose constraints such as lung V20 (‘Vxx’ denotes the percentage of organ volume receiving ≥xx Gy) and mean lung dose (MLD) are considered essential in clinical practice [9–11]. In contrast, clinically significant heart dose parameters reported in the literature span a wide range of dose levels, and no consensus has yet been established [5, 12].

In the era of conventional three-dimensional conformal radiotherapy (3DCRT), the lung was often regarded as the primary at-risk organ. Advances in intensity-modulated radiotherapy (IMRT) and particle therapy have enabled greater sparing of organs at risk beyond the lung. However, when attempting to reduce both lung and heart doses, a trade-off between these organs may arise [13–15]. Several studies have reported the relationship and prognostic significance of lung and heart doses in RT for LA-NSCLC [16, 17]. Nevertheless, limited data are available on which combinations of lung and heart dose parameters exert the greatest prognostic impact. In the present study, we aimed to clarify the combined influence of lung and heart doses on OS through a retrospective multicenter analysis of patients who underwent definitive treatment for LA-NSCLC.

## MATERIALS AND METHODS

### Patient population

We conducted a retrospective analysis using clinical databases from three institutions: Hokkaido University Hospital, NHO Hokkaido Cancer Center and Obihiro Kosei Hospital. The study was approved by the institutional review board (approval number: 021-0178). Eligible patients met the following criteria: (i) diagnosis of primary LA-NSCLC; (ii) clinical stage (cStage) IIIA–IIIC, based on the 8th edition of the Union for International Cancer Control (UICC) TNM classification; and (iii) receipt of RT or CRT with a definitive dose (≥59.4 Gy) between 1 April 2013 and 31 October 2021.

### Treatment

RT was delivered using either 3DCRT or IMRT. Based on preliminary interviews, all institutions generally administered a total dose of 59.4–66 Gy in 30–33 fractions with 1.8–2 Gy per fraction as the standard curative dose. Therefore, this study defined 59.4 Gy or higher as the curative dose. The clinical target volume (CTV) was defined as the gross tumor volume (GTV) plus a uniform 5 mm margin. A planning target volume (PTV) was then created by adding another 5 mm margin around the CTV. Elective nodal irradiation practices varied: one institution omitted it, another consistently included it and the third modified its policy over time. Patients who received durvalumab following CRT were mainly treated after 2018.

### DICOM data collection and contouring organ

Anonymized Digital Imaging and Communications in Medicine (DICOM) and clinical data were obtained from each institution. For each organ at risk (OAR), contours were reviewed by a radiation oncologist with over 5 years of experience. These were assessed for consistency with the thoracic RT OAR atlas proposed by the Radiation Therapy Oncology Group and modified when necessary. Target contours were left unchanged, as originally used at each institution.

### Combined dose distribution calculation and dose-volume histograms analysis

For patients who underwent re-planning during treatment following a new computed tomography (CT) scan, dose distributions were registered to the initial CT using deformable image registration. The total dose was then calculated. Dose-volume histograms (DVHs) were generated for the GTV and each OAR using MIM Maestro (version 7.4.2). The following parameters were extracted: volume, minimum dose, maximum dose, average dose and V5, V10, V20, V30, V40, V50 and V60.

### Assessment of adverse events

Adverse events (AEs) were evaluated using the Common Terminology Criteria for AEs (CTCAE) version 4.0. The onset date of an AE was defined as the date on which the worst grade of the event was first confirmed, measured from the start of RT.

### Statistical analysis

OS was defined as the interval from the start of RT to either confirmed death or last follow-up. OS was estimated using the Kaplan–Meier method, and differences between groups were compared with the log-rank test. The Shapiro–Wilk test assessed the normality of heart and lung dose parameter distributions. Subsequently, Spearman’s rank correlation coefficient was used to evaluate correlations between dose parameters, with coefficients ≥0.6 indicating strong correlation, 0.2 to <0.6 weak correlation and 0 to <0.2 minimal correlation.

For the heart and lung dose parameters, continuous variables were categorized using optimal cutoff values determined by X-tile software [18]. Univariable and multivariable analyses of prognostic factors for OS were performed using the Cox proportional hazards model. Variables with *P*-values <0.1 in univariable analysis were included in the multivariable analysis, along with age, performance status (PS), current smoking history, histologic type, CRT, consolidation durvalumab, GTV volume and cStage. The latter was included despite not meeting statistical significance due to its recognized clinical relevance.

To assess the interaction of lung and heart dose parameters, six lung and eight heart dose combinations were evaluated. For each, patients were divided into two groups: those with both parameters above the cutoff values and the remaining group, with either or both parameters below the cutoff. Associations with OS were assessed using univariable Cox analysis. The combination with the lowest *P*-value was identified as the most promising and included in the multivariable analysis. With a total of 100 events recorded, the model was limited to a maximum of 10 independent variables to maintain an events-per-variable ratio greater than 10 [19]. A *P*-value <0.05 was considered statistically significant. To ensure the robustness of the results, sensitivity analyses were performed to re-evaluate the associations with OS by accounting for the following factors: (a) the impact of missing follow-up data due to early censoring; (b) the influence of heterogeneity in treatment modalities across different time periods; and (c) the effect of inter-institutional heterogeneity. To assess the impact of early censoring, we further conducted analyses by excluding patients with a follow-up period of <12 months. To account for temporal trends, patients were divided into two groups based on the timing of durvalumab approval (before vs after 2018), and this categorization was incorporated as a new covariate in the multivariable analysis. All statistical analyses were performed using JMP Pro (version 17.0.0).

## RESULTS

### Patient outcome

A total of 278 patients met the inclusion criteria and were included in the retrospective analysis: 133 from Institution A, 106 from Institution B and 39 from Institution C. The median follow-up period was 25.3 months (range: 1.6–105 months). Patient characteristics and treatment details are summarized in Table 1. The median patient age was 66 years (range: 34–88 years). Chemotherapy was administered to 243 patients (87.4%), with 6 receiving sequential CRT and 237 receiving concurrent CRT. The irradiation technique was 3DCRT in 234 patients (84.2%) and IMRT (including those who transitioned from 3DCRT) in 44 patients (15.8%).

**Table 1:** Patient and treatment characteristics

	Number or median	% or range
Age (years)	66	34–88
Sex		
Male	207	74.5%
Female	71	25.5%
ECOG Performance status		
0	192	69.0%
≥1	86	31.0%
cStage		
IIIA	153	55.0%
IIIB	96	34.5%
IIIC	29	10.5%
Tumor location		
Right upper	109	39.2%
Left upper	61	21.9%
Left lower	12	4.3%
Other	96	34.6%
Histology		
Adenocarcinoma	139	50.0%
Squamous cell carcinoma	111	39.9%
Other	28	10.1%
Smoke		
Never or ex-smoking	183	65.8%
Current smoking	95	34.2%
Surgical history prior to RT		
No	252	90.6%
Yes	26	9.4%
Chemoradiotherapy		
No	35	12.6%
Yes	243	87.4%
Radiotherapy technique		
3DCRT	234	84.2%
IMRT	44	15.8%
Regional lymph node irradiation		
No	130	46.8%
Yes	148	53.2%
Consolidation durvalumab		
No	172	61.9%
Yes	106	38.1%
Prescription dose (Gy)	60	59.4–70

ECOG = Eastern Cooperative Oncology Group, cStage = clinical stage, RT = radiotherapy, 3DCRT = three-dimensional conformal radiotherapy, IMRT = intensity-modulated radiotherapy, CRT = chemoradiotherapy.

**Fig. 1:**
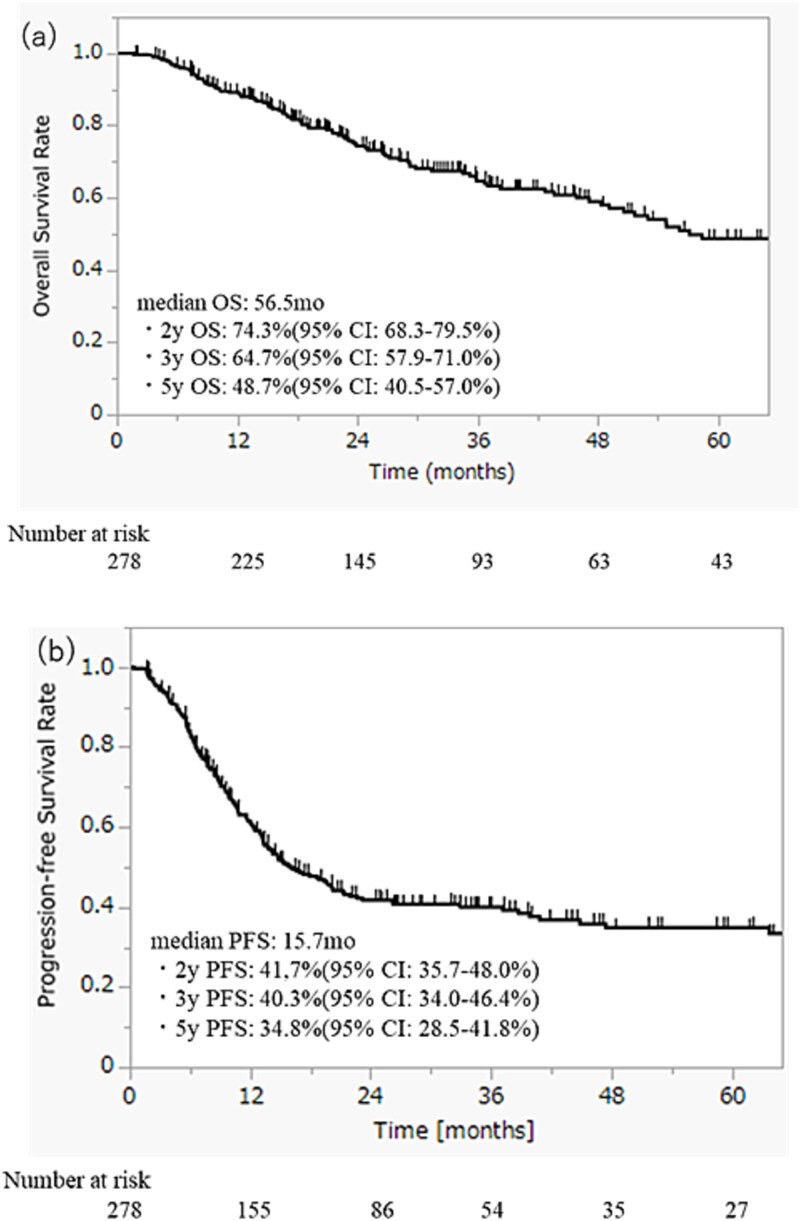
Kaplan–Meier curves of (**a**) overall survival (OS) and (**b**) progression-free survival (PFS) of all patients.

The median OS was 56.5 months. The 2-, 3-, and 5-year OS rates were 74.3% (95% confidence interval: 68.3–79.5%), 64.7% (57.9–71.0%) and 48.7% (40.5–57.0%), respectively. The median PFS was 15.7 months. The 2-, 3- and 5-year PFS rates were 41.7% (35.7–48.0%), 40.3% (34.0–46.4%) and 34.8% (28.5–41.8%), respectively (Fig. 1). A total of 100 mortality events were identified. Causes of death included lung cancer (*n* = 77), other causes (*n* = 17) and undetermined causes (*n* = 6).

### DVHs analysis: correlation between heart and lung dose parameters

Before analyzing correlations, the distribution of each dose parameter was assessed. All heart dose parameters exhibited non-normal distributions, whereas lung dose parameters followed a normal distribution (see Supplementary Table S1). To assess potential multicollinearity between lung and heart variables, the variance inflation factor (VIF) was calculated for each covariate. All VIF values were below 2, indicating the lack of significant multicollinearity [20]. Strong positive correlations were observed within heart and lung dose parameters, respectively. In contrast, heart and lung dose parameters showed low to moderate positive correlations (see Supplementary Fig. S1). In addition, to evaluate potential differences in dose correlations across different RT techniques, color maps were generated for the 3DCRT and IMRT groups. In the IMRT group, correlations within the same organ tended to be slightly lower than those in the 3DCRT group. However, the correlation between the lung and heart doses did not differ significantly between the two techniques (see Supplementary Fig. S2).

**Fig. 2:**
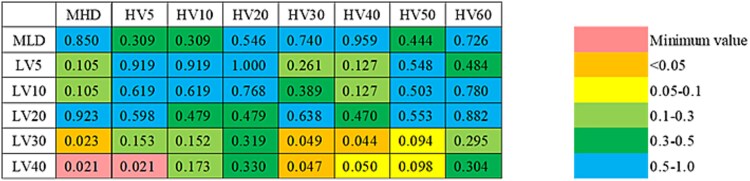
Results of univariable analysis for OS based on combinations of lung and heart dose parameters. Six combinations demonstrated statistical significance. The combinations of MHD/LV40 and HV5/LV40 yielded the lowest *P*-value (*P* = 0.021).

Table 2 presents the threshold values for heart and lung dose parameters based on OS stratification. Univariable analysis was used to evaluate the association between each heart–lung dose parameter combination and OS (Fig. 2). Six combinations demonstrated a statistically significant association with OS (*P* < 0.05), with the mean heart dose (MHD)/lung V40 (LV40) and heart V5 (HV5)/LV40 combinations showing the lowest *P*-value (*P* = 0.021). The thresholds for these dose parameters were MHD ≤24.4 Gy, HV5 ≤63% and LV40 ≤7.7%, respectively. As the patient groups for these two combinations completely overlapped, MHD/LV40 was provisionally selected for multivariable analysis based on overall comparison of *P*-values across other combinations.

**Table 2:** Threshold values for heart and lung dose parameters based on overall survival stratification by X-tile software

		Number	Number	
Dose parameter	Threshold	Low-dose group	High-dose group	*P*-value
MLD	16.9 Gy	241	37	0.066
LV5	42%	207	71	0.453
LV10	35%	212	66	0.308
LV20	29%	223	55	0.096
LV30	8.5%	26	252	0.093
LV40	7.7%	40	238	0.100
MHD	24.4 Gy	247	31	0.031
HV5	63%	249	29	0.153
HV10	56%	246	32	0.181
HV20	47%	248	30	0.364
HV30	36%	250	28	0.064
HV40	31%	249	29	0.057
HV50	19%	201	77	0.107
HV60	15%	245	33	0.295

MLD = mean lung dose, LVxx = the percentage of lung volume receiving ≥xx Gy, MHD = mean heart dose, HVxx = the percentage of heart volume receiving ≥xx Gy, OS = overall survival.

### Toxicity

Pulmonary and cardiovascular AEs are summarized in Tables 3 and 4. Grade ≥3 RP occurred in 10 patients (3.6%), including one grade 5 event. Grade ≥3 cardiovascular events were observed in 11 patients (4.0%), with pericardial effusion being the most common (4 cases). Additionally, three patients experienced grade 5 cardiovascular events: one case each of coronary artery disease, arrhythmia and heart failure. A chi-square test was performed with MHD/LV40 combination as an explanatory variable to assess the incidence of grade ≥3 RP and cardiovascular events. While no statistically significant differences were observed in the incidence of either complication, the high-dose group showed a trend toward a slightly higher incidence of cardiovascular events (see Supplementary Table S2).

**Table 3:** Adverse event: radiation pneumonitis (all grade)

Grade (CTCAE grade)	Number	%	Months from start of RT (median, range)
0	30	10.8	NA
1	136	48.9	4.5 (1.4–23.1)
2	102	36.7	3.7 (0.3–51.0)
3	9	3.2	4 (2.9–17.0)
4	0	0	NA
5	1	0.4	7.4

RT = radiotherapy, CTCAE = Common Terminology Criteria for AEs.

**Table 4:** Adverse event: cardiovascular events (≥grade 3)

Cardiovascular event	Grade 3	Grade 4	Grade 5	Months from start of RT (median, range)
Pericardial effusion	1	3	0	19 (6.1–42.6)
Coronary artery disease	2	0	1	9.5 (3.1–96.3)
Arrythmia	1	0	1	28.5 (Gr.3), 35.9 (Gr.5)
Heart failure	1	0	1	51.5 (Gr.3), 58.4 (Gr.5)

RT = radiotherapy.

### Univariable and multivariable analyses for OS

Univariable Cox analysis identified several predictors of OS (see Supplementary Table S3). Factors with *P*-values <0.1—age, PS, current smoking history, histologic type, CRT, consolidation durvalumab and GTV volume—were included in the multivariable analysis. Multivariable results are shown in Table 5. Independent prognostic factors for OS included: age ≥65 years (Hazard ratio [HR]: 2.06, *P* = 0.002), PS ≥1 (HR: 1.68, *P* = 0.024), histologic type (HR: 2.38, *P* = 0.011), current smoking history (HR: 2.21, *P* < 0.001), CRT (HR: 0.28, *P* < 0.001), GTV volume (HR: 1.43, *P* = 0.007) and the MHD/LV40 combination (HR: 2.35, *P* = 0.003). Sensitivity analyses focusing on the impact of early censoring, temporal heterogeneity in treatment and inter-institutional differences revealed that the results remained consistent even after adjusting for these factors (see Supplementary Tables S4–S6).

**Table 5:** The result of multivariable analysis for overall survival

Variables	HR (95% CI)	*P*-value
Age (years) (≥65 vs <65 years)	2.06 (1.31–3.23)	0.002
PS (≥1 vs 0)	1.68 (1.07–2.62)	0.024
cStage		
IIIA	1 (reference)	
IIIB	1.45 (0.92–2.29)	0.107
IIIC	1.39 (0.70–2.77)	0.352
Histology		
Adenocarcinoma	1 (reference)	
Squamous cell carcinoma	1.12 (0.72–1.74)	0.627
Other	2.38 (1.22–4.66)	0.011
Current smoking	2.21 (1.45–3.38)	<0.001
Chemoradiation	0.28 (0.15–0.52)	<0.001
Consolidation durvalumab	0.72 (0.43–1.21)	0.211
GTV volume (log)	1.43 (1.10–1.85)	0.007
MHD/LV40 combination	2.35 (1.34–4.14)	0.003

PS = performance status, cStage = clinical stage, GTV = gross tumor volume, MHD = mean heart dose, LV40 = percentage of lung volume receiving ≥40 Gy.

## DISCUSSION

Since the RTOG 0617 trial identified heart dose parameters, such as HV5, as significant factors for OS, debate has continued regarding appropriate heart dose constraints in RT for LA-NSCLC [4]. In the long-term analysis of the RTOG 0617 cohort, HV5 remained a negative prognostic factor for OS [21]. While some studies reported no association between heart dose parameters and OS (e.g. Zhang *et al*. [12]), others supported the correlation (e.g. Pan *et al*. [22]). Although no consensus has been reached, parameters such as HV5 and MHD are regarded as promising prognostic indicators.

Although the results for PFS are presented, this study primarily aimed to evaluate the impact of combinations of heart and lung dose parameters on outcomes. Therefore, instead of analyzing PFS, which shows no direct relationship with toxicity in OAR, we focused on analyzing OS, which is considered to have a more direct relationship. A univariable analysis of all 48 heart–lung dose combinations identified six combinations with *P*-values <0.05. The lowest *P*-values were observed for MHD/LV40 and HV5/LV40 (*P* = 0.021). These two groups were identical. The thresholds used for converting continuous variables into categorical variables were determined mechanically; as such, the complete overlap of patient groups between the two combinations is coincidental. However, strong correlations exist among heart DVHs parameters, which increases the likelihood of overlapping high-dose patient groups. Although we conducted multivariable analysis using the MHD/LV40 combination, the issue of multiplicity remains unresolved. Therefore, we cannot definitively conclude that the combination proposed in this study is optimal. Further investigation is warranted to evaluate the significance of other heart DVH parameters—such as MHD and HV5—that have been proposed as prognostic candidates in previous studies. Since the present study cohort predominantly received 3DCRT treatment, cardiopulmonary doses were inevitably influenced by tumor characteristics; thus, DVH parameters may, to some extent, reflect tumor size and localization. However, even within the constraints of 3DCRT, a degree of freedom exists to modulate heart and lung doses independently. In clinical practice, beam arrangements are frequently optimized to spare the heart from high radiation doses. Consequently, understanding the balance of cardiopulmonary doses—which often exist in a trade-off relationship—is a crucial challenge. While the impact of tumor characteristics on prognosis is undeniable, factors related to treatment planning also play a role. Furthermore, several 3DCRT-based studies investigating the association between heart dose and OS evaluated DVH parameters without adjusting for tumor characteristics [8, 23]. Although tumor characteristics and dose distribution are intrinsically linked in any malignancy, it is not uncommon in radiation oncology to discuss the association between OS and DVH as the final output of treatment. We believe that such discussions provide significant clinical value and represent a legitimate and essential area of research within the field.

Watanabe *et al*. developed a combined model composed of heart and lung dose parameters which enabled more accurate risk classification than either set of parameters alone [16]. However, their model consisted solely of dose parameters, and, as they mentioned in the limitations, the influence of clinical background factors was not accounted for. This study confirmed through multivariable analysis including patient background that the MHD/LV40 combination is an independent prognostic factor, and its significance lies in directly evaluating the relationship between dose parameters and OS. Furthermore, many prior studies evaluated dose parameters as continuous variables, making it difficult to apply findings to clinical practice. In this study, dose parameters were treated as categorical variables in the multivariable analysis, thereby allowing the results to be directly applied as dose constraints. Our results indicate that achieving MHD ≤24.4 Gy and LV40 ≤7.7% is significantly associated with superior OS. While these data-driven thresholds are specific to our cohort, they offer valuable preliminary benchmarks for clinical practice. Especially in the absence of an established consensus on integrated heart-lung constraints, these values serve as actionable targets for treatment optimization. Conversely, categorization carries the risk of losing much of the information inherent to the continuous variables, including their non-linearity, potentially reducing the precision of the analysis.

In the present study, durvalumab was not identified as a significant prognostic factor in the multivariable analysis. One contributing factor to this is that 98% of all patients treated after 2018 received durvalumab, while most patients in the non-durvalumab group were treated between 2013 and 2017. Consequently, the durvalumab-treated patient group inevitably included a higher proportion of patients with shorter follow-up periods at the time of analysis, potentially leading to an underestimation of OS, particularly in the early posttreatment period.

In the cohort of this study, only 15.8% of patients received IMRT. However, IMRT is widely used in lung cancer treatment, and approximately half of the patients in RTOG 0617 received IMRT. IMRT enables flexible dose distribution to spare normal tissue, and has been shown to contribute to reducing the risk of severe RP [24]. For lung protection, V20 < 35% and MLD < 20 Gy are key constraints due to their association with RP. For the heart, the National Comprehensive Cancer Network guidelines recommend constraints such as V50 < 25% and MHD < 20 Gy. The results of this study, focusing on the interaction between the heart and lung, reaffirm that heart toxicity should be given equal importance to lung toxicity, which has traditionally been emphasized. This issue cannot be ignored even in the IMRT era. Since further reductions in normal organ doses often involve trade-offs between the heart and lung [15], understanding the combined effects on the heart and lungs is essential for improving the quality of IMRT plans. The data analyzed in this study primarily comprise 3DCRT plans, and caution should be exercised when extrapolating these findings to IMRT owing to its significantly different dose distribution patterns. On the other hand, IMRT dose constraints have evolved from experiences with 3DCRT. For instance, the landmark study by Deasy *et al*. [25], which serves as the basis for the QUANTEC parotid gland constraints, included substantial 3DCRT data yet remains a gold standard in the IMRT era. While our findings are preliminary, we believe they provide valuable insights that may contribute to the establishment of refined dose constraints for the lungs and heart in the future.

**Fig. 3:**
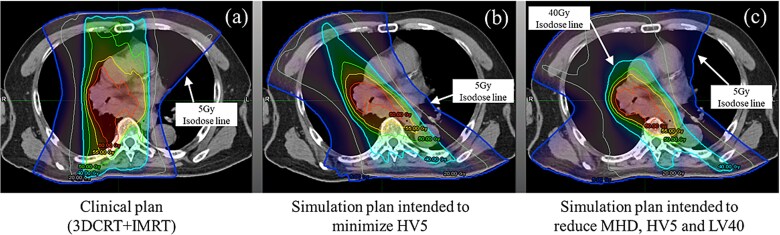
Comparison of dose distribution across the clinical plan and two simulation plans. (**a**) The clinical plan used in actual practice. (**b**) A simulation plan optimized to minimize HV5. (**c**) A simulation plan designed to reduce MHD, HV5 and LV40 simultaneously.

Figure 3 presents a simulation illustrating this trade-off in a single case. The left image shows the clinical plan; the center image displays a plan minimizing HV5 per RTOG 0617 findings. The blue 5 Gy isodose line demonstrates substantial heart dose reduction in the center plan, but with increased high-dose exposure in the right lung. The right image shows a plan that reduces MHD, HV5 and LV40 simultaneously, satisfying our dose constraints (MHD ≤ 24.4 Gy, HV5 ≤ 42% and LV40 ≤ 7.7%). Although the heart area receiving >5 Gy is slightly larger than in the center plan, the right lung’s high-dose region is reduced. It remains uncertain which plan offers better clinical outcomes, but if heart dose reduction is prioritized, a need arises for metrics addressing lung dose balance.

As increasingly stringent dose constraints are required—particularly for the heart—growing attention has been directed toward the potential benefits of proton beam therapy (PBT) for LA-NSCLC. Liao *et al*. reported the results of a prospective randomized trial comparing outcomes of PBT and IMRT in patients with LA-NSCLC [26]. The primary endpoints of the trial were grade ≥3 RP and local failure. However, no statistically significant differences were observed between the two groups for either endpoint. A dosimetric analysis showed that PBT significantly improved heart dose parameters but did not improve lung dose parameters; lung volume receiving ≥20 Gy was higher in the PBT arm. The incidence of RP in the PBT arm was initially estimated to be 5%, but the observed rate was 10.5%. They considered one contributing factor to be the high lung volume exposed to high doses in the PBT arm and emphasized the need for PBT-specific dose constraints. More recently, Yu *et al*. investigated cardiopulmonary toxicity in a comparative analysis of PBT and IMRT, reporting a significant reduction in grade ≥3 RP in the PBT cohort (HR 0.25, *P* = 0.04) [27]. The dosimetric analysis demonstrated that overall dose reductions were achieved not only for the heart but also for the lungs in the PBT group, which likely contributed to the observed reduction in toxicity. Although this study is based on photon beam therapy data and cannot be directly applied to PBT, the conclusion that high-dose lung regions should be considered provides scope for future improvements to PBT treatment planning.

Regarding the dose constraints suggested to be associated with OS in this study, non-compliance with MHD ≤ 24.4 Gy and HV5 ≤ 42% was observed in ~10% of all cases. In contrast, for LV40 ≤ 7.7%, 86% of patients exceeded the threshold. This suggests that LV40 ≤ 7.7% is a particularly stringent dose constraint when applied to 3DCRT planning, partly because such a constraint was not routinely incorporated into treatment planning (Table 2). Since lung volumes exposed to high doses are inherently determined by margins applied to the CTV and PTV, reducing these parameters would require steeper dose gradients at target boundaries. This concept is closely linked to dose conformity in advanced radiation treatment techniques such as IMRT and PBT. As radiation treatment techniques continue to evolve, the present findings highlight the necessity of re-evaluating dosimetric constraints established during the 3DCRT era. The ongoing phase III randomized trial RTOG 1308 (NCT01993810), designed to assess the superiority of PBT over IMRT in LA-NSCLC, does not currently incorporate explicit constraints for high-dose lung volumes. Nevertheless, it is expected that secondary analyses will provide additional insights into dose conformity and other dosimetric parameters in relation to clinical outcomes.

This study has several limitations. First, although the retrospective nature of this study introduces inherent biases, the robustness of our findings was supported by sensitivity analyses yielding consistent results even after accounting for temporal and inter-institutional heterogeneity. Second, the inclusion of patients with relatively short follow-up periods might have influenced the survival outcomes. However, sensitivity analysis excluding patients with <12 months of follow-up yielded consistent results, suggesting that the impact of early censoring on our primary conclusions was minimal. Third, regarding cardiovascular AEs, routine follow-up by cardiology specialists was uncommon, likely leading to underreporting of events, especially those below grade 3 that were not clearly recorded in electronic medical records. However, since the primary endpoint was OS, the impact on core findings is expected to be minimal. Fourth, the identified thresholds (MHD ≤ 24.4 Gy and LV40 ≤ 7.7%) rely on data-driven dichotomization within our cohort. Although these metrics offer actionable guidance for clinicians, they may reflect the specific geometric characteristics of 3DCRT. Consequently, these parameters should be considered preliminary; extensive validation in independent, IMRT-based cohorts is essential to confirm their generalizability and to define robust clinical constraints. Fifth, this study showed insufficient follow-up on cardiovascular events, and the specific mechanisms by which heart toxicity affects OS currently remain unclear. Finally, the definition of curative dose in this study differs from that used in large-scale clinical trials such as the PACIFIC and LAURA trials, which define it as 54 Gy or higher (60 Gy ± 10%). To evaluate the impact of this discrepancy, we performed a post hoc investigation of patients who received doses between 54 and 59.4 Gy (who were initially excluded from the analysis). We identified only two such cases, representing 0.7% of our total cohort. Consequently, even if the curative dose had been defined as 54 Gy or higher, its impact on the overall results would likely be negligible. To resolve these issues, further large-scale retrospective studies conducted in independent cohorts or validation in appropriately designed prospective trials are required to validate these findings.

In conclusion, while the importance of heart dose reduction in LA-NSCLC RT is well established, our results suggest that minimizing high-dose lung exposure (LV40) in conjunction with heart dose may further improve OS. Although these exploratory findings should be interpreted with caution, considering the high-dose lung volume also reflects an increased awareness of the importance of improving target dose conformity and suggests the need to establish novel dose metrics in IMRT and PBT.

## Supplementary Material

Suppementary_Fig_1_rrag034

Supplementary_Fig_2_rrag034

Supplementary_tables_revision_rrag034

## Data Availability

The datasets used and/or analyzed during this study are available from the corresponding author on reasonable request.

## PRESENTATION AT A CONFERENCE

This study was presented in part at the 36th Annual Meeting of the Japanese Society for Radiation Oncology in 2023.
