# Supplementary figures and images for: Optimal combination of heart and lung dose parameters in radiotherapy for locally advanced non-small cell lung carcinoma: a multicenter retrospective study
Source: J Radiat Res. 2026 May 14;67(4):591–9. doi: 10.1093/jrr/rrag034 (PMC13400569; doi:10.1093/jrr/rrag034)

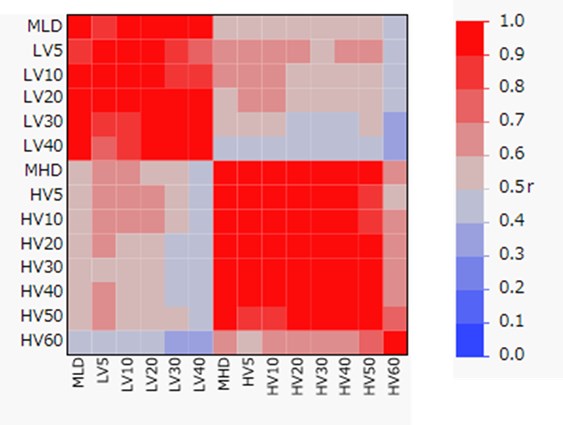

Supplement: Suppementary_Fig_1_rrag034 [file suppementary_fig_1_rrag034.jpeg]

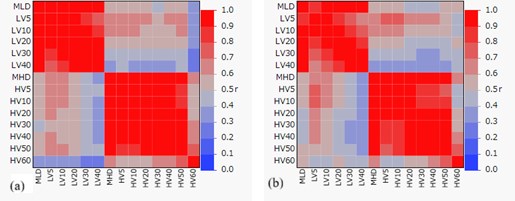

Supplement: Supplementary_Fig_2_rrag034 [file supplementary_fig_2_rrag034.jpeg]
