# Supplementary material for: Optimal combination of heart and lung dose parameters in radiotherapy for locally advanced non-small cell lung carcinoma: a multicenter retrospective study
Source: J Radiat Res. 2026 May 14;67(4):591–9. doi: 10.1093/jrr/rrag034 (PMC13400569; doi:10.1093/jrr/rrag034)
Supplement: Supplementary_tables_revision_rrag034 [file supplementary_tables_revision_rrag034.docx]

Supplementary Table 1

Normality of each dose parameter

|  | Shapiro-Wilk's p-value |
| --- | --- |
| MLD | 0.08 |
| LV5 | 0.51 |
| LV10 | 0.75 |
| LV20 | 0.48 |
| LV30 | 0.1 |
| LV40 | 0.05 |
| MHD | <0.001 |
| HV5 | <0.001 |
| HV10 | <0.001 |
| HV20 | <0.001 |
| HV30 | <0.001 |
| HV40 | <0.001 |
| HV50 | <0.001 |
| HV60 | <0.001 |

MLD= mean lung dose, LVxx= the percentage of lung volume receiving ≥xx Gy, MHD= mean heart dose, HVxx= the percentage of heart volume receiving ≥xx Gy

Supplementary Table 2

1. Chi-square test for two variables: Grade 3 or higher RP and MHD/LV40

|  |  | RP | |  |
| --- | --- | --- | --- | --- |
|  |  | <Gr.3 | ≥Gr.3 | Total |
| MHD/LV40 | Low dose | 241(96.4%) | 9(3.6%) | 250 |
|  | High dose | 27(96.4%) | 1(3.6%) | 28 |
|  | Total | 268 | 10 | P =0.99 |

1. Chi-square test for two variables: Grade 3 or higher cardiovascular event and MHD/LV40

|  |  | Cardiovascular event | |  |
| --- | --- | --- | --- | --- |
|  |  | <Gr.3 | ≥Gr.3 | Total |
| MHD/LV40 | Low dose | 241(96.4%) | 9(3.6%) | 250 |
|  | High dose | 26(92.8%) | 2(7.2%) | 28 |
|  | Total | 267 | 11 | P =0.36 |

RP= radiation pneumonitis, MHD= mean heart dose, LV40= percentage of lung volume receiving ≥40 Gy,

Supplementary Table 3

Univariable analysis for overall survival

|  | Subgroup | HR | 95% CI | p-value |
| --- | --- | --- | --- | --- |
| Age | <65y | 1(reference) |  |  |
|  | ≥65y | 1.65 | 1.10-2.47 | 0.014 |
| Sex | Male | 1(reference) |  |  |
|  | Female | 0.77 | 0.47-1.24 | 0.269 |
| PS | 0 | 1(reference) |  |  |
|  | ≥1 | 1.5 | 0.99-2.26 | 0.060 |
| Tumor site | Other | 1(reference) |  |  |
|  | Right Upper | 1.03 | 0.64-1.65 | 0.905 |
|  | Left Upper | 1.07 | 0.62-1.84 | 0.799 |
|  | Left Lower | 1.69 | 0.74-3.84 | 0.211 |
| cStage | IIIA | 1(reference) |  |  |
|  | IIIB | 1.35 | 0.88-2.06 | 0.164 |
|  | IIIC | 1.5 | 0.79-2.81 | 0.212 |
| Histology | Adeno | 1(reference) |  |  |
|  | Squamous | 1.48 | 0.97-2.26 | 0.066 |
|  | Other | 2.31 | 1.24-4.33 | 0.008 |
| current smoke |  | 1.65 | 1.11-2.45 | 0.013 |
| Surgical history prior to RT |  | 0.87 | 0.40-1.88 | 0.720 |
| CRT |  | 0.39 | 0.24-0.63 | <0.001 |
| RT technique | 3DCRT | 1(reference) |  |  |
|  | IMRT | 0.83 | 0.46-1.48 | 0.520 |
| Regional lymph node irradiation | No | 1(reference) |  |  |
|  | Yes | 0.83 | 0.56-1.23 | 0.364 |
| Consolidation durvalumab |  | 0.65 | 0.40-1.07 | 0.083 |
| Prescription dose |  | 0.99 | 0.93-1.06 | 1.000 |
| GTV volume (Log) |  | 1.28 | 1.04-1.57 | 0.018 |

PS= performance status, cStage= clinical stage, Adeno= adenocarcinoma, Squamous= squamous cell carcinoma, RT= radiotherapy, CRT= chemoradiotherapy, 3DCRT= three-dimensional conformal radiotherapy, IMRT= intensity modulated radiotherapy, GTV= gross tumor volume

Supplementary Table 4

Multivariable analysis for overall survival excluding patients with follow-up periods of less than 12 months

| Variables | HR (95% CI) | p-value |
| --- | --- | --- |
| age (years) (≥65y vs <65) | 2.08 (1.33-3.27) | 0.014 |
| PS (≥1 vs 0) | 1.67 (1.07-2.62) | 0.025 |
| cStage |  |  |
| IIIA | 1 (reference) |  |
| IIIB | 1.45 (0.92-2.28) | 0.112 |
| IIIC | 1.41 (0.70-2.82) | 0.332 |
| Histology |  |  |
| Adenocarcinoma | 1 (reference) |  |
| Squamous cell carcinoma | 1.12 (0.72-1.74) | 0.627 |
| Other | 2.39 (1.22-4.66) | 0.011 |
| Current smoking | 2.20 (1.44-3.36) | <0.001 |
| Chemoradiation | 0.28 (0.15-0.52) | <0.001 |
| Consolidation durvalumab | 0.75 (0.44-1.26) | 0.275 |
| GTV volume (log) | 1.42 (1.10-1.85) | 0.008 |
| MHD/LV40 combination | 2.31 (1.31-4.07) | 0.004 |

PS= performance status, cStage= clinical stage, GTV= gross tumor volume, MHD= mean heart dose, LV40= percentage of lung volume receiving ≥40 Gy

Supplementary Table 5

Multivariable analysis for overall survival: Sensitivity analysis for temporal changes in clinical practice

| Variables | HR (95% CI) | p-value |
| --- | --- | --- |
| Year treatment started (≥2018 vs <2018) | 1.02(0.55-1.90) | 0.939 |
| age (years) (≥65y vs <65) | 1.99(1.26-3.14) | 0.002 |
| PS (≥1 vs 0) | 1.67(1.07-2.62) | 0.024 |
| cStage |  |  |
| IIIA | 1 (reference) |  |
| IIIB | 1.45(0.92-2.29) | 0.109 |
| IIIC | 1.38(0.69-2.78) | 0.363 |
| Histology |  |  |
| Adenocarcinoma | 1 (reference) |  |
| Squamous cell carcinoma | 1.12(0.72-1.74) | 0.627 |
| Other | 2.38(1.21-4.67) | 0.012 |
| Current smoking | 2.21(1.45-3.38) | <0.001 |
| Chemoradiation | 0.28(0.15-0.52) | <0.001 |
| Consolidation durvalumab | 0.71(0.36-1.37) | 0.302 |
| GTV volume (log) | 1.43(1.10-1.85) | 0.007 |
| MHD/LV40 combination | 2.35(1.34-4.14) | 0.003 |

PS= performance status, cStage= clinical stage, GTV= gross tumor volume, MHD= mean heart dose, LV40= percentage of lung volume receiving ≥40 Gy

Supplementary Table 6

Multivariable analysis for overall survival: Sensitivity analysis for inter-institutional differendes

| Variables | HR (95% CI) | p-value |
| --- | --- | --- |
| Institution |  |  |
| Site A | 1 (reference) |  |
| Site B | 0.80(0.50-1.28) | 0.343 |
| Site C | 1.57(0.72-3.43) | 0.259 |
| age (years) (≥65y vs <65) | 2.11(1.34-3.32) | 0.001 |
| PS (≥1 vs 0) | 1.82(1.15-2.88) | 0.013 |
| cStage |  |  |
| IIIA | 1 (reference) |  |
| IIIB | 1.44(0.91-2.27) | 0.116 |
| IIIC | 1.29(0.64-2.58) | 0.472 |
| Histology |  |  |
| Adenocarcinoma | 1 (reference) |  |
| Squamous cell carcinoma | 1.15(0.73-1.82) | 0.544 |
| Other | 2.41(1.24-4.72) | 0.01 |
| Current smoking | 2.17(1.40-3.37) | <0.001 |
| Chemoradiation | 0.28(0.15-0.52) | <0.001 |
| Consolidation durvalumab | 0.62(0.35-1.07) | 0.082 |
| GTV volume (log) | 1.46(1.13-1.92) | 0.004 |
| MHD/LV40 combination | 2.20(1.23-3.91) | 0.012 |

PS= performance status, cStage= clinical stage, GTV= gross tumor volume, MHD= mean heart dose, LV40= percentage of lung volume receiving ≥40 Gy
